# A one health glossary to support communication and information exchange between the human health, animal health and food safety sectors

**DOI:** 10.1016/j.onehlt.2021.100263

**Published:** 2021-05-08

**Authors:** Tasja Buschhardt, Taras Günther, Taran Skjerdal, Mia Torpdahl, Jörn Gethmann, Maria-Eleni Filippitzi, Catharina Maassen, Solveig Jore, Johanne Ellis-Iversen, Matthias Filter, The OHEJP Glossary Team

**Affiliations:** a German Federal Institute for Risk Assessment, Department of Biological Safety, Max-Dohrn-Str. 8-10, 10589 Berlin, Germany; b Norwegian Veterinary Institute, Arboretveien 57, N-1433 Ås, Norway; c Statens Serum Institut, 5 Artillerivej, DK-2300 Copenhagen S, Denmark; d Friedrich-Loeffler-Institut – Federal Research Institute for Animal Health (FLI), Südufer 10, D-17493 Greifswald-Insel Riems, Germany; e Sciensano, Juliette Wytsmanstraat 14, 1050 Elsene, Belgium; f National Institute for Public Health and the Environment, Antonie van Leeuwenhoeklaan 9, 3721 MA Bilthoven, the Netherlands; g Norwegian Institute of Public Health, Lovisenberggata 8, 0456 Oslo, Norway; h National Food Institute at the Technical University of Denmark, Kemitorvet 202, DK-2800 Kgs Lyngby, Denmark

**Keywords:** Surveillance, Definition of terms, Dictionary, Scientific terminology, FAIR data, Virtual research environment

## Abstract

Collaboration across sectors, disciplines and countries is a key concept to achieve the overarching One Health (OH) objective for better human, animal and environmental health. Differences in terminology and interpretation of terms are still a significant hurdle for cross-sectoral information exchange and collaboration within the area of OH including One Health Surveillance (OHS). The development of the here described glossary is a collaborative effort of three projects funded within the One Health European Joint Programme (OHEJP). We describe the infrastructure of the OHEJP Glossary, as well as the methodology to create such a cross-sectoral web resource in a collaborative manner. The new OHEJP Glossary allows OH actors to identify terms with different or shared interpretation across sectors. Being aware of such differences in terminology will help overcome communication hurdles in the future and consequently support collaboration and a more inclusive development of OHS. The OHEJP Glossary was implemented as a web-based, user-friendly and searchable infrastructure that complies with the Findable, Accessible, Interoperable, Reusable (FAIR) data principles. Maintenance, enrichment and quality control of the OHEJP Glossary is supported through a flexible and updatable curation infrastructure. This increases the uptake potential and exploitation of the OHEJP Glossary by other OH initiatives or tools and services.

## Highlights

• Cross-sector communication is a key prerequisite of the One Health (OH) approach.
• Differences in terminology and definitions hinder cross-sector communication.
• The OHEJP^1^ Glossary supports OH efforts and helps to overcome communication hurdles.
• The OHEJP Glossary is a web-based, searchable, user-friendly, and FAIR^2^ resource.
• A dedicated curation infrastructure supports content enrichment and quality control.

## Graphical abstract

**Figure:**
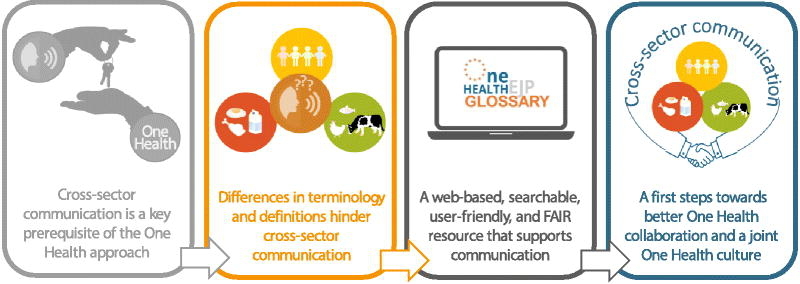

## 1 Introduction

The World Health Organization (WHO) has defined One Health (OH) as an approach, to design and implement programmes, policies, legislation and research, where multiple sectors communicate and work together to achieve better public health outcomes [1]. This approach recognizes that many health problems, especially those related to zoonotic agents and antimicrobial resistance, are multi-sectoral and require expertise and action from several disciplines. Sectors such as public health, animal health, plant health and the environment should be included in the OH approach. Other OH definitions also highlight that collaboration across sectors is a key concept to achieve the overarching OH objective [[2], [3], [4], [5]]. The needs and benefits of cross-sectoral collaboration to address public and animal health problems are apparent [6,7], but traditions, as well as cultural and financial differences between sectors still represent significant hurdles. Moreover, problems regarding responsibilities, data or information sharing and privacy restrictions present barriers for efficient and unimpeded collaboration. Furthermore, fundamental things such as scientific terminology and definitions have developed independently within each sector over time, which complicates cross-sectoral communication and collaboration until today.

In 2018, the One Health European Joint Programme (OHEJP) started with the ambition to support the practical implementation of the OH framework in Europe [8]. This program supported the establishment of new activities and OH resources via so-called “integrative action” and “joint research” projects, such as ORION [9], NOVA [10] and COHESIVE [11]. In a joint effort these three projects teamed up to develop a cross-sectoral glossary (OHEJP Glossary) that could help to improve mutual understanding of terms of terms used in different OH sectors and disciplines. The OHEJP Glossary should include relevant terms from the three sectors, namely public health, food safety and animal health. It should further allow, where available, to highlight alignments, but also provide sector specific definitions of terms. To ensure broad acceptability and future adoption, the new OHEJP Glossary was created on the basis of existing resources from international organizations such as European Food Safety Authority (EFSA), European Centre for Disease Control (ECDC), WHO, World Organization for Animal Health (OIE), Food and Agriculture Organization of the United Nations (FAO), Centers for Disease Control and Prevention of the United States (CDC), and Codex Alimentarius. Furthermore, it was implemented as a user-friendly and searchable web resource that complies with the FAIR data principles [12]. Twenty-two national agencies and institutes from twelve European countries supported the collaborative and cross-sectoral development process of the new OHEJP Glossary. In this article, the OHEJP Glossary is introduced together with the methodology that was implemented to establish this true cross-sectoral, community driven OH resource.

## 2 Materials and methods

The OHEJP Glossary has been developed in three phases. During the requirement analysis phase, the end user needs, the scope and technical constraints were defined. During the development phase, the necessary methodology and glossary content was created in an iterative and agile manner. The maintenance phase started with the development of the FAIR OHEJP Glossary website for end users and continues with regular updates on the glossary content and technical infrastructure. Terms and definitions related to the development of the OHEJP Glossary are listed in Appendix Table A.

### 2.1 Requirement analysis

The initial requirements were defined through a set of dedicated (web-)meetings carried out within and between the European Joint Programme (EJP) projects ORION, NOVA and COHESIVE. Experts from three OH sectors (public health, food safety, animal health) participated in these meetings. Requirements collected initially within the three projects were finally merged across projects. One of the first tasks during the requirement phase was to identify glossaries that cover topics of OH and assess whether available glossaries were FAIR and supporting the OH community in the communication problems related to differences in terminology. Over the course of the agile glossary development process, new feature requests emerged that were continuously documented and implemented. Knowledge categories were e.g. introduced that supported a more structured selection of terms. Each knowledge category was defined as an overarching area or topic relevant to OH (see also Appendix Table B). By providing these knowledge categories OHEP experts were guided on which OH areas they should provide terms for and it supported the goal that all of these categories were covered for each OH sector.

A main outcome of this phase was the agreement on the glossary term selection criteria, which were specified as follows (1–4):

1. The term was relevant for at least one of the above mentioned three OH sectors in Europe
2. A definition for the term by an internationally recognized organization (e.g. EFSA, ECDC, WHO, etc.) or other trusted OH sources (e.g. research projects, scientific journal) was available. Terms with no such definition were only included if they were relevant to more than one OH sector or the scope of the three EJP projects.
3. Selected terms belonged to one of the following knowledge categories, which had previously been defined (Appendix Table B)
  a. Sampling and study design used in OH surveillance
  b. Epidemiology in relation to OH
  c. EU regulations related to OH
  d. Dissemination of OH outcomes
  e. Laboratory, analytical, bioinformatics or statistical methods used in OH surveillance
  f. Project tasks and resources relevant to the EJP projects NOVA, ORION or COHESIVE
4. The terms and definitions existed in English

### 2.2 Development phase

During the development phase work was performed within two main areas:

1. The creation of glossary content, which in itself comprised of four steps: identification, annotation, curation and enrichment
2. The establishment of software resources to support the teams of OHEJP glossary editors and curators

The identification of terms that are compliant with the selection criteria was performed by groups of experts from each sector through screening of web resources, documents or guidelines from internationally recognized organizations e.g. EFSA, ECDC, WHO, FAO, European Commission (EC). Other terms with particular relevance for OH were collected from other research projects or initiatives e.g. Risk-Based Animal Health Surveillance Systems (RiskSur) [13], Network for Evaluation of One Health (NEOH) [14], European Network for Neglected Vectors and Vector-Borne Infections (EurNegVec) [15]. In cases where no official definition could be identified, alternative sources of definitions e.g. from company websites, dictionaries were adopted or new definitions were generated by the corresponding expert group and subjected to review by the OHEJP Glossary curators (hereafter referred to as curators). Further, the members of each group provided terms which, based on their own experience, were particularly used in their country, sector and/or in general in the OH context. In the annotation step, glossary editors provided definitions for each term and referenced those accordingly. Glossary editors also performed quality control, i.e. double-checking that the definition provided for each term matched the given reference and that information provided on the reference itself was correct and sufficiently detailed. In cases where multiple definitions for the very same term were available, each of those definitions were included as independent items into the OHEJP Glossary.

During the curation and enrichment step, a cross-sectoral team of glossary curators (at least two experts from each sector) performed the following tasks for each glossary item:

1. Specified whether a term is used in their sector and, if this was the case, whether the given definition was appropriate. Alternatively, they indicated that they were unable to identify an adequate definition for that term in their sector.
2. Assessed whether a definition was “shared” between the OH sectors or was “specific” to one sector.
3. Annotated each term with the belonging knowledge categories (tags): “Data processing and analysis”, “Epidemiology”, “Sampling and laboratory testing”, etc.
4. If glossary curators identified a mismatch between a given official definition and their own work experience, they could compose a new definition that was labelled as “adapted version of a given definition”.
5. Uncertainties and disagreements during the curation rounds were documented and resolved by consulting additional glossary curators from the three OHEJP projects.

To support the content creation and curation, a specifically designed shared Google Sheet [16] was developed. Specifically, adapted features like conditional formatting and an integrated data item form supported glossary editors and glossary curators.

### 2.3 Maintenance phase

To establish a FAIR, user-friendly and maintainable OHEJP Glossary web resource for end users, it was decided to build upon a so-called Virtual Research Environment (VRE) that was developed within the AGINFRA+ project [17]. Within this VRE, the glossary content could be made accessible via the web interface of the VRE Data Catalogue component. For efficient long-term administration and curation of the glossary content it was, however, necessary to develop customized web applications. Here, the open-source software KNIME Analytics Platform [18] and the KNIME Server infrastructure of the German Federal Institute for Risk Assessment (BfR) was used. Dedicated KNIME-based web services were developed to reduce the workload for glossary editors and curators. This customized software backend also enables the automatic integration of other web resources, like the ORION Zotero [19] reference manager group (https://www.zotero.org/groups/2344323/orion/items). A detailed description of this technical infrastructure will be published elsewhere.

## 3 Results

### 3.1 Requirement analysis

The joined assessment of OH experts from the ORION, COHESIVE and NOVA projects proved that existing glossaries (like those listed in Appendix Table C) were not sufficient to identify conflicting or shared definitions for terms commonly used by different OH sectors. The existing glossaries were either too focused on one discipline (e.g. sequencing), had only marginal coverage of cross-sectoral terms or were not available as a FAIR resource. This led to the development of the inclusive and FAIR OHEJP Glossary that incorporates relevant terms from the public health, animal health and the food sector and highlighted where terms and interpretation aligned and differed between the three sectors. Although the already existing glossaries do not fulfil the above-mentioned criteria, they proved as very useful sources for specific definitions for the OJEHP Glossary.

### 3.2 OHEJP glossary infrastructure

**Fig. 1:**
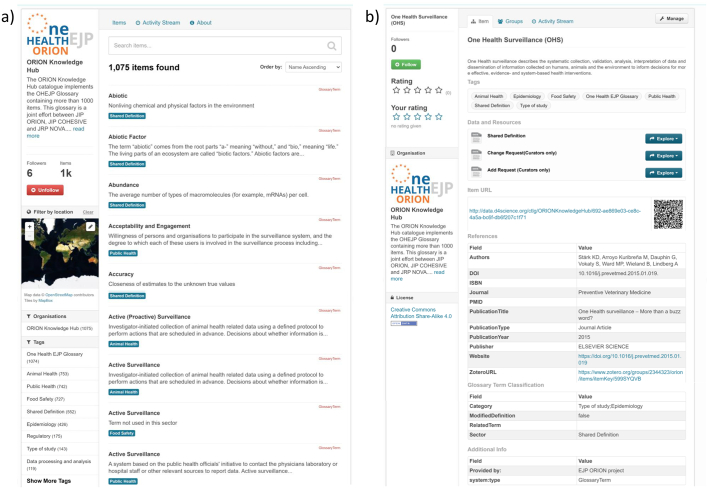
Screenshots of the OHEJP Glossary web resource. The landing page (a) has a search bar at the top and tags (in the left column) can be used to filter the OHEJP Glossary items. The assigned One Health sector for each item (term + definition + metadata) is shown in a blue box below the item (see also Appendix Table B). The detailed view of each OHEJP Glossary item is exemplary shown for the term One Health Surveillance (b). This includes the complete definition, assigned tags, the Item URL (with a QR code), reference information and information on whether the definition was adapted from the original definition given in the reference. OHEJP Glossary users can also make a change or add request (under data and resources) or rate the term (top left). (For interpretation of the references to colour in this figure legend, the reader is referred to the web version of this article.)

The OHEJP Glossary and its linked resources are accessible via the following website: https://foodrisklabs.bfr.bund.de/ohejp-Glossary/. The OHEJP Glossary is hosted on a VRE within the AGINFRA+ gateway and integrates links to dedicated web services for glossary curators and editors. The OHEJP Glossary (Fig. 1) allows end users to perform a full-text search or a search by title with a specific syntax. Users can also filter the glossary content by “tags” that are based on the knowledge category or OH sector assignment. As terms are frequently defined differently in different sectors, the OHEJP Glossary holds multiple items for several terms. Each glossary item has its own dedicated website, through a so-called Item Uniform Resource Locator (URL), where all available metadata is presented, e.g. the definition, the reference for the definition, the tags given by the glossary curators and even comments or ratings from users. The availability of a specific Item URL makes it possible to refer unambiguously to a specific definition of a term or version of an item. Due to the VRE backend, the OHEJP Glossary fulfils the FAIR data principles as it can also be accessed via an open software interface (API). Registered VRE users have the option to issue a “change or add request” for terms and definitions and to comment or rate glossary items. The functionalities and features of the OHEJP Glossary are further demonstrated in a webinar that can be accessed here: https://data.d4science.net/nPk4.

**Fig. 2:**
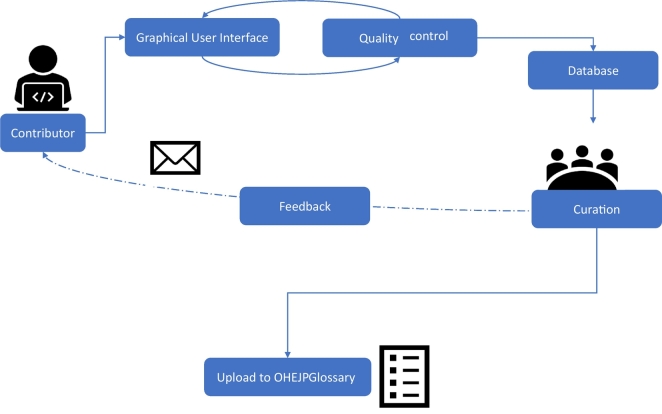
Schematic representation of the technical infrastructure established for OHEJP Glossary content generation, curation and quality control. OHEJP Glossary users can issue a change request (to request a change in a definition or metadata) or add request (to add a new OHEJP Glossary item with a corresponding term, definition and metadata) through the OHEJP Glossary user interface. The request is curated by the OHEJP glossary curators and (if accepted) the change or new item is uploaded to the OHEJ Glossary.

In addition, a glossary curation backend infrastructure (Fig. 2) has been designed to give utmost support to the OHEJP experts in all content generation and curation tasks. Based on the experiences from the content development phase, the required manual work of editors and curators is sometimes error-prone and therefore needs best possible technical assistance to achieve a sufficiently high data quality (e.g. to highlight incomplete references and sector-assignments) for the OHEJP Glossary.

### 3.3 General findings

**Fig. 3:**
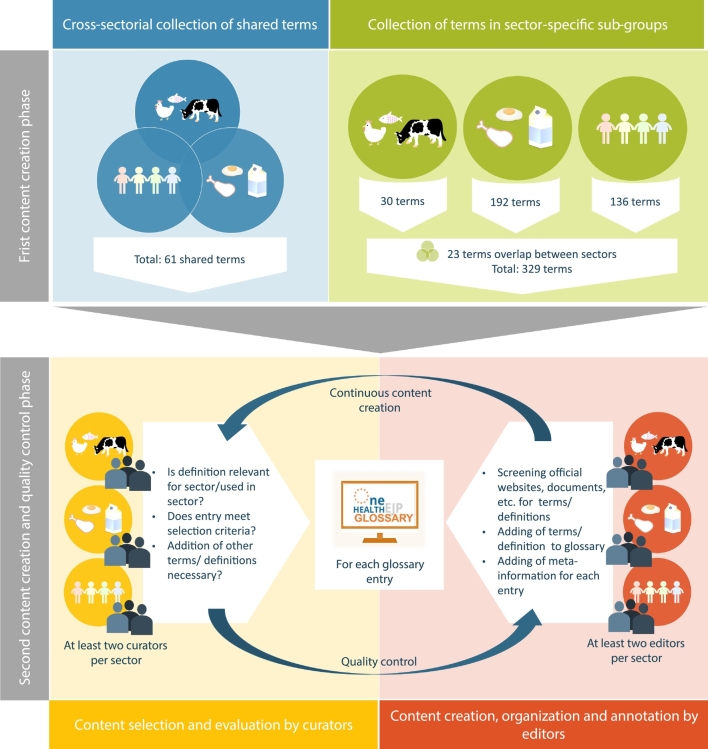
Overview of Glossary development and curation process and phases. The content creation was performed in two phases. In the first phase (top: blue and green box) content generation was performed in two ways, in a first approach a team of experts from all three OH sectors (animal health, public health and food safety) collected relevant terms together (blue box), while a second approach was to collect terms in small sector-specific groups of experts (green box). The second approach resulted in a much higher number of collected terms. The second content generation phase (bottom: yellow and red box) was combined with content curation by at least two experts per OH sector (yellow box) and quality control by OHEJP editors (red box). The decision to combine content development and data curation in one step helped to restrict the workload for the glossary team to an acceptable level. (For interpretation of the references to colour in this figure legend, the reader is referred to the web version of this article.)

The generation of content for the OHEJP Glossary is a continuously ongoing process. In the first iteration round, the initial cross-sectoral expert group from the ORION project identified only 61 candidate terms for inclusion into the OHEJP Glossary (Fig. 3). This number of terms increased substantially from 61 to 390, when the expert group was split up into sector-specific sub-groups to collect terms from their sector-specific perspective. The sector-specific expert groups for animal health, food safety and public health identified 30, 192 and 136 candidate terms, respectively. When merged, only 23 terms overlapped between all the sectors. Despite this, the sector-centred procedure proved efficient to create a joint cross-sectoral resource. Considering that, after this initial creation of the glossary content, the curation for each item was performed by glossary curators of at least two experts from each sector (Fig. 3). The overlap of terms between sectors increased thereby with the ongoing curation process.

The defined scope, target audience and functionality of the OHEJP Glossary influenced not only the selection of terms, but also influenced the content generation and curation procedures that had to be implemented. Specifically, the objective to create a resource that helps to identify aligned or divergent definitions across the three OH sectors made it necessary to spend significant efforts on a content enrichment process. The decision to perform this content enrichment together with the data curation step helped to restrict the workload for the glossary team to an acceptable level. In turn, the available knowledge category and OH sector assignment for each term now gives a more explicit meaning and creates added value for the end users, as they can filter by those tags as well. The knowledge category assignment is particularly valuable when the same term is used in different contexts e.g. the term “analytical method” is used in the context of OHS data analysis but also in a laboratory context.

Finally, the joint collaborative work on the OHEJP Glossary content led to the realization that the identified cross-sectoral (shared) definitions are not the only asset of the OHEJP Glossary. As the experience from the glossary expert groups with members from different sectors repeatedly showed, it is often impossible to agree on a consensus definition for specific terms. It became apparent that term definitions are frequently neither aligned within one sector, nor on national level or between international stakeholders. As a consequence, the OHEJP Glossary evolved into an open resource that provides “all” available definitions and can now identify terms where misunderstanding due to conflicting or non-aligned definition of terms might occur. One example for this is the term “Active Surveillance” (enter title:”Active Surveillance” in search field at https://aginfra.d4science.org/web/orionknowledgehub/catalogue).

### 3.4 OHEJP glossary content

As of summer 2020, the OHEJP Glossary contained over 1000 curated items that covered 694 unique terms. The majority of terms and corresponding definitions were labelled as “shared between sectors” (574 items), despite initial discrepancies of terms shared between sectors. Of definitions only shared by two sectors, the animal and food sector shared definitions for 45 terms, while public health and animal health shared definitions for 19 terms. Definitions for four glossary terms were shared between public health and food safety only. Sector specific definitions, with no corresponding definition in other sectors were 79, 67 and 92 for animal health, food safety and public health, respectively.

## 4 Discussion

Collaboration and communication between actors from different sectors, disciplines and countries is the foundation for OH surveillance and other OH activities. The collaborative work within OHEJP has shown that the risk of misunderstandings increases when experts from different OH sectors are brought together. An important underlying reason for this is the independent development of different “languages” and the perceived meaning of terms within each sector. One specific problem is that OH related documents (e.g. reports and guidelines) rarely contain glossaries with an integrative list of OH terms and their specific meaning within the context of the given document. By not including a glossary, the authors assume that all readers, including those from other OH sectors, know the meaning and anticipate the same interpretation of the terms used. Consequently, this can lead to misunderstandings and misinterpretation of the documents itself, but also of the related data. The same potential for misunderstandings and misinterpretation holds true for verbal cross-sectoral communication, if the interpretations of terms have not previously been clarified.

The OHEJP Glossary was developed to support cross-sectoral communication and collaboration. It serves as a readily available “translation tool” for the effective communication between the corresponding sectors animal health, public health and food safety. It is anticipated that the OHEJP Glossary can promote a positive communication experience for experts and stakeholders from different OH sectors, disciplines and countries. If misunderstandings are avoided, OH actors are also enabled to profit from each other's knowledge, experience and data, which will conceivably foster a more inclusive development of OH activities in the future. Without the willingness and ability of different OH actors to communicate unambiguously, OH systems will remain sectoral and fragmented. The adoption and dissemination of the OHEJP Glossary by experts and stakeholders from different OH sectors, disciplines and countries are thereby considered as first steps towards better OH collaboration and the successive generation of a joint OH culture.

The OHEJP Glossary could become a true OH building block, because it is designed as a continuously updateable, cross-sectoral community resource. Currently, the OHEJP Glossary enables users to look up OH related terminology, find currently used definitions with complete references and even allows to reference each glossary item directly via a persistent term-specific resource identifier (Item URL). It also helps to identify definitions that are shared between the public health, animal health and food safety sector or terms with divergent sector-specific interpretation. As the OHEJP Glossary is implemented as a FAIR web resource, it is able to interface with other software tools and increases the uptake potential by other initiatives or projects.

Currently, the OHEJP Glossary only contains a collection of terms that were considered relevant for any of the three sectors: animal health (including wildlife-health), food safety and public health involved in the OHEJP. Extending the scope to the environmental or social sciences is a future opportunity achievable by the technical infrastructure and the methodological approach that both allow extensions to other OH disciplines or sectors. The inclusion of the environmental sector would be of outstanding relevance. This sector is often neglected in OH initiatives [20], despite being an important aspect in tackling the global warming and AMR challenges, where more integrated activities of animal/human/plant health and environmental sciences are required [[21], [22], [23], [24]]. Other useful features, like the support for multiple languages or the integration of “uncurated” terms and definitions, i.e. items lacking the sector and knowledge category metadata, could be easily accomplished with the current technical framework.

The development of the OHEJP Glossary fostered three “lessons learned” on how to overcome some generic OH challenges:

1. A dedicated cross-sectoral collaboration activity is required to initiate the development of critical OH community resources. The project funding from the OHEJP provided a unique opportunity for the development of the OHEJP Glossary.
2. A quality-controlled content generation and enrichment will remain a challenge in the future. Despite the availability of online glossaries from official sources (Appendix Table C) content generation remains a labour-intensive manual work, as not all relevant information is provided in a FAIR way. In many cases definitions are even hidden in EU directives or regulations, technical reports, standards or guidelines. Most difficulties were encountered with definitions for laboratory (e.g. for laboratory methods) and bioinformatics terms. Another significant amount of work is linked with the ambition to enrich glossary items with additional metadata where multiple rounds of content review by several OH experts are required. In these review rounds partly diverging requests from different glossary curators or conflicting opinions need to be solved. In this respect, the strategy to allow the inclusion of different definitions for one term instead of forcing the creation of consensus definitions proved to be very successful.
3. A community-driven FAIR OHEJP Glossary requires appropriate software tools that can support the collaborative content generation and curation process. Existing tools for ontology or thesaurus generation like VocBench [25] were unfortunately not user-friendly enough to be used. An elaborated open source infrastructure is now available that supports all OHEJP Glossary related management and quality control steps, while offering a transparent, intuitive user interface for all end user needs.

## 5 Conclusion

For the OHEJP Glossary to create impact, it needs to be used and jointly maintained by researchers, institutes and agencies inside the EU. An even greater impact and inclusivity could be reached if the OHEJP Glossary was used outside the EU as well. This is a challenge, as it implies that, first, a critical awareness of this new OH community resource among relevant stakeholders must be established; and then, agencies and institutions might need to change some of their established routines and long-standing traditions in relation to communication and definitions of terms. Nevertheless, there is reason for optimism as the innovative technical OHEJP Glossary infrastructure provides several unique features for end users, e.g. a FAIR source for definitions of terms and their correct references as well as a persistent Item URL, that could be used to reference definitions (and versions thereof) in user-specific documents or reports. The established technical infrastructure would also allow functional extension, e.g. to support automatic translation of terms, incorporation of definitions from different languages or the extension into other OH sectors like the environmental sector. The future maintenance of the OHEJP glossary is not yet settled. One desirable scenario would be that European agencies could take the lead in the community-driven maintenance of the OHEJP Glossary. An even wider strategy for the dissemination and maintenance could be a collaborative effort by the authors of the Tripartite Zoonoses Guide and European agencies. In the meantime, the OHEJP program will serve the dissemination and maintenance tasks.

## Funding

The 10.13039/501100007083ORION project is part of the European Joint Programme One Health EJP, which has received funding from the European Union's Horizon 2020 Research and Innovation Programme under Grant Agreement No 773830.

## Author contributions

Tasja Buschhardt: Conceptualization, Methodology, Investigation, Data Curation, Visualization, Writing - Original Draft. Taras Günther: Methodology, Software, Formal Analysis, Visualization, Writing - Review & Editing. Taran Skjerdal: Investigation, Data Curation, Conceptualization, Writing - Review & Editing. Mia Torpdahl: Investigation, Data Curation, Conceptualization, Writing - Review & Editing. Jörn Gehtmann: Investigation, Data Curation, Conceptualization, Writing - Review & Editing. Maria-Eleni Filippitzi: Investigation, Data Curation, Conceptualization, Writing - Review & Editing. Catharina Maassen: Conceptualization, Writing - Review & Editing. Solveig Jore: Investigation, Data Curation, Conceptualization, Writing - Review & Editing. Johanne Ellis-Iversen: Investigation, Data Curation, Conceptualization, Writing - Review & Editing. Matthias Filter: Conceptualization, Resources, Software, Funding acquisition, Writing - Original Draft. The OHEJP Glossary curation team: Investigation, Data curation, Conceptualization.

## Declaration of Competing Interest

None.

## Appendix A Supplementary data

Supplementary material Image 1
