# Supplementary material for: A one health glossary to support communication and information exchange between the human health, animal health and food safety sectors
Source: One Health. 2021 May 8;13:100263. doi: 10.1016/j.onehlt.2021.100263 (PMC8141924; doi:10.1016/j.onehlt.2021.100263)
Supplement: Supplementary file 1 — Supplementary material [file mmc1.docx]

# Appendix A

Appendix Table A: Glossary of terms used within the OHEJP Glossary manuscript. The focus of this glossary is not on One Health, but rather on terms used in the development and the description of the OHEJP Glossary and the technical infrastructure.

| **Term** | **Definition** | **Online reference** | **OHEJP Glossary Item URL** |
| --- | --- | --- | --- |
| Curation | See curators |  |  |
| Curation infrastructure | An information technology (IT) infrastructure (including e.g. hardware, software, the internet) used to provide data curation and enforce data curation policies. | OHEJP Term^a^ | <http://data.d4science.org/ctlg/ORIONKnowledgeHub/0c999220-d10c-43fb-81ed-8705a116e14b> |
| Curators | Curators ensure that the data are fit for contemporary purpose and available for discovery and reuse. They are responsible for the data management, which includes (a) data acquisition, data integration, or reuse of existing data, (b) review of data quality; (c) provision of standard-compliant metadata, including references. | OHEJP Term^a^ | <http://data.d4science.org/ctlg/ORIONKnowledgeHub/7bb181a6-418e-459f-b9c8-425ae957228a> |
| Data Catalogue | A curated collection of metadata about datasets and their data elements. | <https://casrai.org/rdm-glossary/> | <http://data.d4science.org/ctlg/ORIONKnowledgeHub/0704a773-a086-45de-a562-01ee2eb36ecc> |
| Data element | A unit of data for which the definition, identification, representation (term used to represent it), and permissible values are specified by means of a set of attributes. | <https://casrai.org/rdm-glossary/> | <http://data.d4science.org/ctlg/ORIONKnowledgeHub/355826fc-6e5f-4e13-a9e6-76da0f2a4277> |
| Data quality | The reliability and application efficiency of data. It is a perception or an assessment of dataset’s fitness to serve its purpose in a given context. Aspects of data quality include: Accuracy, Completeness, Update status, Relevance, Consistency across data sources, Reliability, Appropriate presentation, Accessibility. Within an organization, acceptable data quality is crucial to operational and transactional processes and to the reliability of analytics, business intelligence, and reporting. Data quality is affected by the way data are entered, stored and managed. Maintaining data quality requires going through the data periodically and scrubbing it. Typically this involves updating, standardizing, and de-duplicating records to create a single view of the data, even if it is stored in multiple disparate systems. Data quality assurance (DQA) is the process of verifying the reliability and effectiveness of data. | <https://casrai.org/rdm-glossary/> | <http://data.d4science.org/ctlg/ORIONKnowledgeHub/d15e6c1d-41ed-449c-9ca5-89f3e6f96cbf> |
| Editing | See editors |  |  |
| Editors | Editors support curators in data management process. Their main task is data acquisition and metadata review. They do not assess whether the data are fit for purpose. | OHEJP Term^a^ | <http://data.d4science.org/ctlg/ORIONKnowledgeHub/ee7668a8-dcec-4526-a617-6f40d50df535> |
| Item | See data element |  |  |
| FAIR data | Findable, accessible, interoperable and re-usable data, i.e. data that are managed according to the FAIR Guiding Principles. The FAIR Principles describe distinct considerations for contemporary data publishing environments with respect to supporting both manual and automated deposition, exploration, sharing, and reuse. | <https://www.nature.com/articles/sdata201618> | <http://data.d4science.org/ctlg/ORIONKnowledgeHub/18-2312c17a-430f-4862-a849-6538fdafe9be> |
| Requirements | Features of a program, system, dataset, or product that are quantifiable, detailed, and relevant to the specified end use. | <https://casrai.org/rdm-glossary/> | <http://data.d4science.org/ctlg/ORIONKnowledgeHub/8b1c86a4-d2e7-43f0-b703-570ea5f93418> |
| Virtual Research Environment (VRE) | A VRE comprises a set of online tools and other network resources and technologies interoperating with each other to facilitate or enhance the processes of research practitioners within and across institutional boundaries. A key characteristic of a VRE is that it facilitates collaboration amongst researchers and research teams providing them with more effective means of collaboratively collecting, manipulating and managing data, as well as collaborative knowledge creation. | <https://www.jisc.ac.uk/guides/implementing-a-virtual-research-environment-vre> | <http://data.d4science.org/ctlg/ORIONKnowledgeHub/155-6cdadb1b-fed6-4ce4-9efd-35cb37871aa3> |
| Web resource | 1. Addressable units of information that are addressed through Uniform Resource Identifiers (URIs). 2. The early notion of static addressable documents or files has evolved to a more generic and abstract definition. Every ‘thing’ or entity that can be identified, named, addressed or handled in any way whatsoever in the web at large or in any networked information system. Examples include: an electronic document or data stored on the Web, an image, a service (e.g., “a weather report), a collection of other resources. Each resource must have a URI. | <https://casrai.org/rdm-glossary/> | <http://data.d4science.org/ctlg/ORIONKnowledgeHub/2e7178ec-a951-461a-8cba-d54324de75cb> |

^a^ No suitable online reference available. Term was defined by OHEJP Glossary curators.

# Appendix B

Appendix Table B: Definition of OHEJP Glossary tags. Each OHEJP Glossary item is assigned to a One Health sector (SE) and a One Health knowledge category (KC). This supports the filtering of OHEJP Glossary items and allows to specify the meaning of each term in an explicit OH context.

| **Type of tag** | **Name of tag** | **Definition** |
| --- | --- | --- |
| SE | Animal Health | Terms and definitions related to the part of society working to ensure a state of physical and psychological well-being of animals including wildlife health and maintenance of efficient production systems contributing to food safety and food security. |
| SE | Public Health | Terms and definitions related to the art and science of dealing with the protection and improvement of community health by organized community effort and including preventive medicine and sanitary and social health, as well as the science of protecting and promoting the Health of individuals and the communities where they live. |
| SE | Food Safety | Terms and definitions related to the assurance that food will not cause harm to the consumer when it is prepared and/or eaten according to its intended use. |
| SE | Shared Definition | Terms and definitions shared between the Animal Health, Public Health and Food Safety sector |
| SE | Environment | Terms and definitions related to all that is external to the individual, including physical, biological, social, cultural and other factors. |
| KC | Type of study | Terms and definitions related to the type of study that is performed e.g. clinical investigation, monitoring, survey, surveillance et cetera. |
| KC | Epidemiology | Terms and definitions related to the determinants, incidences, distribution, exposure, and possible control of diseases and other health-related states or events. Terms related to occurrence research, which study and quantify the relationship between determinants and the occurrence of an illness in a population. |
| KC | Sampling and laboratory testing | Terms and definitions related to data collection (sampling), laboratory analysis and results thereof, e.g. methods, equipment, and sampling plans, sampling units, variables to be measured and results of laboratory tests. |
| KC | Regulatory | Terms and definitions related to regulatory institutions, authorities, regulations, guidelines, legal requirements and laws. |
| KC | Data processing and analysis | Terms and definitions that describe surveillance data processing and analysis e.g. cleaning, validation, integration, anonymization of data or methods for statistical analysis or comparison of data, as well as, bioinformatics. This also includes processing of metadata and developments to improve data harmonization. |
| KC | Dissemination | Terms and definitions related to data dissemination and evaluation e.g. reporting obligations, reporting formats, databases for reporting, and dissemination infrastructures. |
| KC | ORION: One health suRveillance Initiative on harmOnization of data collection and interpretatioN Term | Terms and definitions used in the ORION project, such as terms that are not “common knowledge” and have been used in the project proposal, deliverables, presentations, publications, etcetera. The ORION project aims at establishing and strengthening inter-institutional collaboration and transdisciplinary knowledge transfer in the area of surveillance data integration and interpretation, along the One Health (OH) objective of improving health and well-being [9]. |
| KC | NOVA: Novel approaches for design and evaluation of cost-effective surveillance across the food chain Term | Terms and definitions used in the NOVA project, such as terms that are not “common knowledge” and have been used in the project proposal, deliverables, presentations, publications, etcetera. The NOVA project strives to develop new surveillance tools and methods and to harmonise and optimise the use of existing surveillance system data [10]. |
| KC | COHESIVE: One Health Structure In Europe Term | Terms and definitions used in the COHESIVE project, such as terms that are not “common knowledge” and have been used in the project proposal, deliverables, presentations, publications, etcetera. The COHESIVE project aims at strengthening/improving (structured) collaboration between the human and veterinary domain in the area of risk‐analysis of (emerging) zoonoses in European Member States [11]. |
| KC | Others | Terms and definitions that do not comply with any of the categories above. |

# Appendix C

Appendix Table A : List of available glossaries from official organisations and/ or research projects. The type pf glossary is specified and the overarching focus and/or context of the glossaries is summarized. The glossaries can be accessed or downloaded through the provided URL.

| **Type of glossary** | **Organisation/ project** | | **Title of glossary / document** | | **URL** | | **Overarching focus/context** |  |
| --- | --- | --- | --- | --- | --- | --- | --- | --- |
| E-Book | - | | Dictionary of Epidemiology | | <http://www.oxfordreference.com/view/10.1093/acref/9780199976720.001.0001/acref-9780199976720> | | epidemiology, biostatistics, public health, medicine |  |
| Webpage | CASRAI^[[1]](#footnote-1)^ | | Research Data Management Glossary | | <https://casrai.org/rdm-glossary/> | | reserach data management |  |
| Webpage | CDC^[[2]](#footnote-2)^ | | Epidemiology Glossary | | <https://www.cdc.gov/reproductivehealth/data_stats/glossary.html> | | reproductive health, data and statistics |  |
| Webpage | CDC | | Glossary of Terms Related to Antibiotic Resistance | | <https://www.cdc.gov/narms/resources/glossary.html> | | antibiotic resistance, outbreak investigation, public health, animal health, fodd safety |  |
| Webpage | CDC | | Principles of Epidemiology in Public Health Practice | | <https://www.cdc.gov/csels/dsepd/ss1978/glossary.html> | | epidemiology, public health, biostatistics |  |
| Webpage | Codex Alimentarius (FAO^[[3]](#footnote-3)^ & WHO^[[4]](#footnote-4)^) | | Glossary of Terms | | <http://www.fao.org/fao-who-codexalimentarius/codex-texts/dbs/vetdrugs/glossary/en/> | | Residues of Veterinary Drugs in Foods |  |
| Webpage | DDI^[[5]](#footnote-5)^ | | DDI Glossary | | <https://www.ddialliance.org/resources/ddi-glossary> | | DDI-specific glossary, metadata |  |
| Webpage | DNA Link Sequencing Lab | | Glossary of Sequencing terms | | <https://www.dnalinkseqlab.com/glossary/> | | Sequencing, bioinformatics, molecular biology |  |
| Excel for download | ECDC^[[6]](#footnote-6)^ | | [TESSy^[[7]](#footnote-7)^ metadata report](https://www.ecdc.europa.eu/en/publications-data/tessy-metadata-report) | | <https://www.ecdc.europa.eu/en/publications-data/tessy-metadata-report> | | public health surveillance |  |
| Webpage | EFSA^[[8]](#footnote-8)^ | | Glossary | | <https://www.efsa.europa.eu/en/glossary-taxonomy-terms> | | risk assessment, food safety |  |
| Excel for download | EFSA | | EFSA data collection framework (DCF) | | https://zenodo.org/record/3243215#.XudzGEUzZaR | | Food safety, surveillance, monitoring |  |
| Webpage | EU | | IATE^[[9]](#footnote-9)^ European Union terminology | | <https://iate.europa.eu/home> | | EU-specific terminology |  |
| Webpage | EU | | EU Vocabularies | | <https://publications.europa.eu/en/web/eu-vocabularies/home> | | EU-specific terminology |  |
| PDF for download | EurNEgVEc^[[10]](#footnote-10)^ | | EurNEgVEc One Health Dictionary | | https://www.eurnegvec.org/publications/other/EurNegVecDictionary.pdf | | Vectors, Vectore-Borne Infections, One Health |  |
| Webpage | EUROSTAT^[[11]](#footnote-11)^ | | Eurostat glossary | | <https://ec.europa.eu/eurostat/statistics-explained/index.php/Thematic_glossaries> | | General and regional statistics, Economy and finance, Population and social conditions, Industry and services, Agriculture, forestry and fisheries, International trade, Transport, Environment and energy, Science, technology and digital society |  |
| Webpage | FAO | | FAO TERM PORTAL | | <http://www.fao.org/faoterm/en/> | | Food safety, food security, etc. |  |
| PDF for download | FAO/WHO/OIE^[[12]](#footnote-12)^ | | Tripartite guide on zoonotic diseases recently published by FAO, WHO and OIE | | <https://www.oie.int/fileadmin/Home/eng/Media_Center/docs/EN_TripartiteZoonosesGuide_webversion.pdf> | | One Health, zoonotic disease |  |
| Webpage | FDA^[[13]](#footnote-13)^ | | Glossary of terms | | <https://www.fda.gov/patients/clinical-trials-what-patients-need-know/glossary-terms> | | Clinical trials |  |
| Webpage | National Human Genome Research Institute | | Talking Glossary of Genetic Terms | | [https://www.genome.gov//genetics-glossary/m#glossary](https://www.genome.gov/genetics-glossary/m#glossary ) | | Genetics |  |
| PDF for download | (NEOH^[[14]](#footnote-14)^) | | Integrated approaches to health | | <https://www.wageningenacademic.com/doi/epdf/10.3920/978-90-8686-875-9> | | One Health |  |
| Webpage | OECD^[[15]](#footnote-15)^ | | OECD Glossary of Statistical Terms | | <http://stats.oecd.org/glossary/> | | Statistics |  |
| Webpage | OIE | | Terrestrial Animal Health Code-Glossary | | [http://www.oie.int/index.php?id=169&L=0&htmfile=glossaire.htm#terme_zoonose](http://www.oie.int/index.php?id=169&L=0&htmfile=glossaire.htm#terme_zoonose  ) | | Animal Health, Surveillance |  |
| PDF for download | OIE | | OIE - Terrestrial Animal Health Code | | <http://www.oie.int/fileadmin/Home/eng/Health_standards/tahc/current/glossaire.pdf> | | Animal Health, Surveillance, Risk Analysis, Disease Control, International Trade |  |
| PDF for download | OIE | | OIE Manual of Diagnostic Tests and Vaccines for Terrestrial Animals 2019 | | <http://www.oie.int/fileadmin/Home/eng/Health_standards/tahm/0.06_GLOSSARY.pdf> | | Animal Health, Diagnostic Tests, Vaccines |  |
| PDF for download | RISKSUR^[[16]](#footnote-16)^ consortium | | Glossary | | <https://fp7-risksur.eu/terminology/glossary> | | Animal Health, Surveillance |  |
| Webpage | SDMX^[[17]](#footnote-17)^ | | SDMX Glossary | | <https://sdmx.org/wp-content/uploads/SDMX_Glossary_Version_2_0_October_2018.htm> | | data and metadata exchange |  |
| Webpage | UN^[[18]](#footnote-18)^ | | UN Terms | | <https://unterm.un.org/unterm/portal/welcome> | | subjects relevant to the work of the United Nations |  |
| PDF for download | UNECE^[[19]](#footnote-19)^ | | Glossary of terms on statistical data editing | | <https://webgate.ec.europa.eu/fpfis/mwikis/essvalidserv/images/3/37/UN_editing_glossary.pdf> | | Statistics |  |
| PDF for download | UNECE | | Terminology on statistical metadata | | <http://ec.europa.eu/eurostat/ramon/coded_files/UNECE_TERMINOLOGY_STAT_METADATA_2000_EN.pdf> | | Statistics, Metadata |  |
| PDF for download | UNODC^[[20]](#footnote-20)^ | | Glossary of Terms for Quality Assurance and Good Laboratory Practices | | <https://www.unodc.org/documents/scientific/ST_NAR_26_E.pdf> | | Quality Assurance, Laboratory Practices |  |
| PDF for download | US Department of Commerce | | A Glossary of Standards-Related Terminology | | <https://www.nist.gov/document/glossary-standards-related-terminology> | | standardization, certification, laboratory accreditation, quality control |  |
| PDF for download | USDA^[[21]](#footnote-21)^/APHIS^[[22]](#footnote-22)^/Veterinary Services | | Glossary of terms for surveillance standards | | <https://www.aphis.usda.gov/vs/nahss/docs/surveillance_standards_v1_full_doc.pdf> | | Animal Health, Surveillance, Data Standards |  |
| Webpage | USDA | | Data Management Glossary | | <https://www.nal.usda.gov/main/data/data-management-glossary> | | data management, data curation |  |
| PDF for download | WHO | | THE WHO Health Promotion Glossary | | <https://www.who.int/healthpromotion/about/HPG/en/> | | Health Promotion |  |
| Webpage | WHO | | International Clinical Trials Registry Platform Glossary | | <https://www.who.int/ictrp/glossary/en/> | | clinical trials |  |
|  | |  | |  | |  | | |

1. CASRAI: Consortia Advancing Standards in Research Administration Information [↑](#footnote-ref-1)
2. CDC: Centers for Disease Control and Prevention [↑](#footnote-ref-2)
3. FAO: Food and Agriculture Organization of the United Nations [↑](#footnote-ref-3)
4. WHO: World Health Organization [↑](#footnote-ref-4)
5. DDI: Data Documentation Initiative [↑](#footnote-ref-5)
6. ECDC: European Centre for Disease Prevention and Control [↑](#footnote-ref-6)
7. TESSy: The European Surveillance System [↑](#footnote-ref-7)
8. EFSA: European Food Safety Authority [↑](#footnote-ref-8)
9. IATE: Interactive Terminology for Europe [↑](#footnote-ref-9)
10. EurNEgVEc: European Network for Neglected Vectors and Vector-Borne Infections [↑](#footnote-ref-10)
11. EUROSTAT: European Statistical Office [↑](#footnote-ref-11)
12. OIE: World Organisation for Animal Health [↑](#footnote-ref-12)
13. FDA: U.S. Food and Drug Administration [↑](#footnote-ref-13)
14. NEOH: Network for Evaluation of One Health [↑](#footnote-ref-14)
15. OECD: Organisation for Economic Co-operation and Development [↑](#footnote-ref-15)
16. RISKSUR: Risk-Based Animal Health Surveillance Systems [↑](#footnote-ref-16)
17. SDMX: Statistical Data and Metadata Exchange [↑](#footnote-ref-17)
18. UN: United Nations [↑](#footnote-ref-18)
19. UNECE: United Nations Economic Commission for Europe [↑](#footnote-ref-19)
20. UNODC: United Nations Office on Drugs and Crime [↑](#footnote-ref-20)
21. USDA: United States Department of Agriculture [↑](#footnote-ref-21)
22. APHIS: Animal and Plant Health Inspection Service [↑](#footnote-ref-22)
